# Reproducibility and accuracy of corneal curvature measurements in patients with and without dry eye: a device-based study

**DOI:** 10.3389/fmed.2025.1565740

**Published:** 2025-05-16

**Authors:** Xuemei Zhang, Kunhong Xiao, Taichen Lai, Rongyong Zhang, Mengxue Huang, Ying Xue, Li Li, Huiying Rao

**Affiliations:** ^1^Shengli Clinical Medical College of Fujian Medical University, Fujian Provincial Hospital, Fuzhou University Affiliated Provincial Hospital, Fuzhou, China; ^2^Department of Ophthalmology, Fuzhou University Affiliated Provincial Hospital, Fuzhou University, Fuzhou, China; ^3^Department of Optometry, School of Medical Technology and Engineering, Fujian Medical University, Fuzhou, China

**Keywords:** dry eye, reproducibility, multiple device, corneal curvature, tear film

## Abstract

**Purpose:**

To evaluate the reproducibility and accuracy of corneal curvature measurements using five different devices in patients with and without dry eye, and to identify the most reliable device for clinical use in dry eye patients.

**Methods:**

This study included 68 eyes from dry eye patients (dry eye group) and 48 eyes from non- dry eye patients (non- dry eye group). Corneal curvature was measured with five devices: Corneal topography, ARK-1a, IOL Master 700, OPD-Scan III, Pentacam. A total of 580 examination reports were collected. Reproducibility was assessed using intraclass correlation coefficients (ICCs), and accuracy was evaluated using Bland-Altman analyses. Differences between devices were examined through paired-sample *t*-tests or Wilcoxon signed-rank tests.

**Results:**

In both groups, the ICCs for ARK-1a, IOL Master 700, OPD-Scan III, and Pentacam were all > 0.9, indicating high reproducibility. Compared to the dry eye group, the non- dry eye group generally had narrower confidence intervals. Bland-Altman analyses showed that the 95% limits of agreement (LOA) were wider in the dry eye group, indicating greater variability. However, the LOA of IOL Master 700 and OPD-Scan III remained below 1.0 in the dry eye group, suggesting higher reliability and potential clinical advantages.

**Conclusion:**

Dry eye significantly affects the reliability of corneal curvature measurements, especially with optical reflection-based devices. Corneal topography and Pentacam are more sensitive to tear film abnormalities, while the IOL Master 700 and OPD-Scan III show more consistent results, making them preferable for clinical practice, such as clinical applications including refractive surgery planning, contact lens fitting, and preoperative cataract assessment.

## 1 Introduction

Approximately two-thirds of the eye’s refractive power is provided by the cornea (1). Its refractive power, shape, and optical quality have a direct impact on both the eye’s refractive state and visual performance. Corneal curvature measurements are broadly utilized in a range of settings, including refractive surgery, orthokeratology lens fitting, myopia management, and clinical diagnostics (2). In refractive surgery, corneal curvature serves as a critical factor in refractive evaluation, surgical design, and postoperative outcome assessment (3–5). Likewise, in orthokeratology and contact lens fitting, precise characterization of corneal curvature is essential for ensuring proper lens alignment and improving wearer comfort, while reducing corneal irritation and potential infections (6, 7). Regular monitoring of corneal curvature is also fundamental for tracking disease progression and treatment outcomes. Moreover, alterations in corneal curvature are clinically pertinent indicators for the early detection of corneal disorders such as keratoconus, as well as for the evaluation of refractive errors and myopia prevention (8). Accurate measurement and longitudinal follow-up of corneal curvature are thus vital aspects of effective ophthalmic care and the prevention of vision-threatening complications. With the widespread use of electronic devices, screen time has significantly increased, leading to a dramatic rise in the prevalence of dry eye (9, 10). In Asia, the current prevalence of dry eye syndrome ranges from approximately 20.1% (11). According to the latest definition proposed by the TFOS DEWS II: “Dry eye is a multifactorial disease of the ocular surface characterized by a loss of homeostasis of the tear film, and accompanied by ocular symptoms, in which tear film instability and hyperosmolarity, ocular surface inflammation and damage, and neurosensory abnormalities play etiological roles.” This definition highlights the importance of tear film instability and visual impairment (12). It highlights the importance of assessing Tear Film Breakup Time (TFBUT) in diagnosing and managing dry eye. Given that the tear film constitutes the eye’s primary refractive interface, maintaining its smoothness and stability is critical to achieving clear vision (13). When measuring corneal curvature, the tear film serves as the reflective surface; instability or disruption of this film often leads to corneal surface irregularities, dryness, and microdamage. Such changes may alter corneal curvature and compromise the accuracy of measurements made by ophthalmic instruments (14). Therefore, maintaining tear film stability is crucial not only for obtaining accurate corneal curvature measurements but also for ensuring their consistency. In recent years, various studies have attempted to elucidate the impact of dry eye on corneal curvature measurements (15). Recent studies have shown that dry eye can affect the reliability of corneal curvature measurements prior to cataract surgery (16). Dry eye Patients exhibit greater variability in mean corneal curvature (K) and corneal astigmatism (17). The average non-invasive break-up time (NIBUT) test value is also known to affect mean K and corneal astigmatism (18).

Currently, various clinical devices are available for measuring corneal curvature, and the repeatability and consistency of some devices have been validated. However, there are few reports on their accuracy, precision, and consistency in dry eye patients (14, 19). This research gap complicates the development of standardized protocols for measuring corneal curvature in dry eye patients, who often exhibit tear film instability and irregularities of the ocular surface. Moreover, there is a paucity of systematic research on selecting appropriate measurement devices to improve the consistency and accuracy of corneal curvature data in this population. Addressing this shortfall is essential for enhancing diagnostic reliability and optimizing patient outcomes. Comparative investigations involving multiple devices can systematically elucidate their respective performances in dry eye, enabling clinicians to identify devices that yield more stable and reliable corneal measurements. The five devices chosen for this study–Corneal topography, ARK-1a, IOL Master 700, OPD-Scan III, and Pentacam–were selected due to their widespread clinical application and representation of distinct corneal curvature measurement methodologies. Corneal topography and Pentacam, utilizing Placido ring and Scheimpflug imaging technologies, respectively, are particularly vulnerable to tear film instability; The ARK-1a device uses autorefractor technology and is potentially influenced by ocular accommodation; IOL Master 700 (employing optical coherence tomography) and OPD-Scan III (based on wavefront aberrometry) are theoretically less susceptible to tear film variations. Therefore, comparing measurements from these five devices in dry eye and non-dry eye patients can more comprehensively evaluate the impact of dry eye on different measurement technologies. The primary objective of this study is to assess corneal curvature measurement outcomes across these five devices in dry eye and non- dry eye patients, thereby offering evidence-based guidance for clinicians regarding optimal device selection in dry eye management.

## 2 Materials and methods

### 2.1 Study subjects and grouping

This study enrolled patients who visited the Department of Ophthalmology at Fujian Provincial Hospital between May 2024 and January 2025.

All patients underwent examination with a Keratograph 5M ocular surface analyzer (Oculus GmbH, Wetzlar, Germany). To measure tear film breakup time (TFBUT), patients were instructed to position their chin on the instrument’s chin rest and press their forehead against the support while gazing straight ahead. After focusing on the tear film, patients were asked to blink twice, then to keep their eyes open as long as possible. Once they could no longer keep their eyes open, they were instructed to blink or close their eyes, enabling recording of the TFBUT. Patients with NIBUT (non-invasive breakup time) ≥ 10 s were assigned to the non-dry eye group, while those with NIBUT < 10 s were placed in the dry eye group. A total of 580 examination reports were collected, yielding 68 eyes in the dry eye group and 48 eyes in the non- dry eye group.

This study was approved by the Ethics Committee of Fujian Provincial Hospital (K202412014) and conducted in accordance with the principles outlined in the Declaration of Helsinki. Written informed consent was obtained from all participants prior to enrollment, and clinical examinations were performed by experienced ophthalmologists.

Inclusion criteria were: (1) Age ≥ 18 years and able to cooperate in completing this study. (2) Discontinuation of soft contact lenses for two weeks or more. Discontinuation of rigid contact lenses for one month or more.

Exclusion criteria: (1) Use of ocular or systemic medication within the past month. (2) Ocular diseases such as keratoconus, corneal scarring, malnutrition, conjunctivitis, blepharitis, and pterygium. (3) History of ocular trauma or surgery.

### 2.2 Optical principles and methods of corneal curvature measurement

#### 2.2.1 Corneal topography in the Oculus Keratograph 5M (Oculus GmbH, Wetzlar, Germany)

Corneal topography is performed by projecting a Placido ring pattern onto the cornea and capturing its reflected image, thus assessing the corneal surface based on optical reflection principles. The Placido ring features multiple concentric circles that reflect off the corneal surface, with any corneal irregularities producing local changes in the reflected image. For measurement, the device is set to the appropriate height, and the patient’s chin and forehead are stabilized on the instrument supports. The patient focuses on a central fixation light to ensure uniform corneal reflection. After several blinks, the technician aligns the device according to the on-screen prompts and acquires an automatic image once the ideal focal position is achieved.

#### 2.2.2 Auto Ref/Keratometer ARK-1a(Nidek)

The ARK-1a uses the Mire Ring technique to measure corneal curvature, minimizing artifacts attributable to eyelid movements and thereby enhancing measurement accuracy. The patient positions their forehead and chin on the designated supports. After aligning the measurement lens with the pupil center and adjusting focus, the device automatically calculates the refractive power and corneal curvature parameters. Repeat measurements three times and take the average value; measurement results are considered reliable when the diopter error ≤ 0.5 D and axis error < 3° across three measurements.

#### 2.2.3 IOL Master 700 (Carl Zeiss Meditec AG, Jena, Germany)

The IOL Master 700 employs partial coherence interferometry and image-processing algorithms to measure the distance between six reflected light spots arranged symmetrically on the anterior corneal surface (diameter: 2.3 mm). By determining the separation between these spots, the device computes the radius of curvature on the annular corneal surface as well as mean corneal curvature between two points. During the examination, the patient’s chin and forehead are placed on the respective supports while they look at a central fixation light. Six light spots must be sharply focused. The patient is asked to blink to maintain tear film stability before each reading. Measurements are repeated five times, and the average is used for final analysis. A green “√” indicator and distortion-free corneal curvature image confirm adequate measurement quality.

#### 2.2.4 OPD-Scan III (Nidek Co., Ltd., Gamagori, Japan)

The OPD-Scan III integrates Placido disk technology and dynamic retinal scanning. It projects structured light (e.g., concentric rings) onto the corneal surface and captures the reflected image through a lens system. Deformation in the rings is then analyzed to calculate corneal curvature across different locations. Measurements are taken in a darkroom, with the patient’s forehead and chin secured. Following standardized operating protocols for the OPD-Scan III, the patient is advised to blink to preserve tear film integrity. The technician aligns the focus following on-screen guidance, captures the image automatically or manually, and saves the measurement when the Placido ring count exceeds 21. A 4-mm pupil diameter is selected for data analysis.

#### 2.2.5 Pentacam (Oculus Optikgeräte GmbH, Wetzlar, Germany)

Pentacam is a non-contact ophthalmic imaging system based on the Scheimpflug principle. One camera rotates around the eye to build a three-dimensional model of the anterior segment, including corneal curvature data for different diameters, while a second camera tracks eye movements to correct for misalignment. During the examination, the patient’s chin and forehead are positioned on the instrument supports in a darkroom, and the patient looks at a fixation target. After focusing, the patient blinks to hydrate the cornea, and an automatic measurement is initiated. Only scans meeting the “OK” criteria in Pentacam’s Quality Specification (QS) system were included in this study. If yellow or red warnings appear (such as blinking, eye movement, or light interference), remeasurement is required.

### 2.3 Data collection

Each participant underwent corneal curvature measurement with the five devices in the same predefined sequence. Each device was operated by an experienced technician masked to the aims of the study. All technical personnel received standardized training, including equipment operation procedures, measurement techniques, and how to identify and avoid measurement errors. Before the study began, all technical personnel were required to pass consistency tests to ensure their operational level met standards. To prevent measurement sequence from affecting results, this study adopted randomized measurement sequences. The interval between measurements on each device was controlled within 10 min to reduce the impact of natural tear film fluctuations on measurement results. Three separate measurements were collected per device, and the mean values were used to obtain K1, K2, and astigmatism. No ophthalmic medications were administered before or during testing. Relevant demographic data (e.g., age and sex) were retrieved from electronic medical records.

### 2.4 Statistical analysis

Data were processed using R (version 4.4.1). The Shapiro–Wilk test was used to assess data normality. Between-group comparisons of baseline characteristics were performed using either the *t*-test or Wilcoxon rank-sum test, depending on data distribution. Categorical variables were compared with the chi-square test. Pairwise consistency between instruments in both groups was evaluated using intraclass correlation coefficients (ICCs) and Bland–Altman plots. Differences in K1, K2, and astigmatism among devices within each group were examined using paired-sample *t*-tests or Wilcoxon signed-rank tests. Statistical significance was defined as a *p*-value < 0.05.

## 3 Results

### 3.1 Baseline characteristics and corneal curvature of participants

A total of 68 eyes with dry eye and 48 eyes without dry eye were included in this study. The gender composition was comparable between the two groups, but the mean age of the dry eye group was significantly higher. Regarding corneal curvature, K1 and K2 values in the dry eye group were significantly higher than those in the non- dry eye group for most devices (such as corneal topography, ARK-1a, and IOL Master 700). However, there were no significant differences in astigmatism values across all devices. Different devices showed varying levels of significance in detecting intergroup differences, suggesting that dry eye condition may affect the accuracy of corneal curvature measurements and that device selection could influence the results (Table 1).

**TABLE 1:** The baseline characteristics between the dry eye group and non-dry eye group.

Characteristic	dry eye *N* = 68^1^	non-dry eye *N* = 48^1^	*p*-value^2^
**Sex**			> 0.9
Female	48 (71%)	34 (71%)	
Male	20 (29%)	14 (29%)	
Age	73 (70, 77)	69 (62, 74)	0.002
**Corneal topography**			
K1	44.74 ± 1.34	44.08 ± 1.70	0.028
K2	44.43 ± 1.36	43.71 ± 1.61	0.014
Astigmatism	0.75 (0.45, 1.20)	0.75 (0.35, 1.20)	0.8
**ARK-1a**			
K1	44.15 ± 1.16	43.51 ± 1.57	0.018
K2	45.07 ± 1.31	44.53 ± 1.53	0.055
Astigmatism	0.75 (0.50, 1.25)	0.75 (0.50, 1.25)	> 0.9
**IOL Master 700**			
K1	44.21 ± 1.31	43.42 ± 1.52	0.004
K2	45.11 ± 1.37	44.47 ± 1.52	0.023
Astigmatism	0.70 (0.47, 1.30)	0.82 (0.45, 1.38)	0.5
**OPD-Scan III**			
K1	44.19 ± 1.27	43.631.83	0.074
K2	45.05 ± 1.33	44.57 ± 1.76	0.11
Astigmatism	0.78 (0.44, 1.14)	0.65 (0.38, 1.17)	0.7
**Pentacam**			
K1	44.25 ± 1.26	43.75 ± 1.92	0.12
K2	45.04 ± 1.35	44.63 ± 1.91	0.2

^1^*n* (%); median (Q1, Q3); mean ± SD;

^2^Pearson’s chi-squared test; Wilcoxon rank sum test; two sample *t*-test.

### 3.2 Two-by-two comparison of inter-device measurement consistency in the dry eye and non- dry eye groups

**FIGURE 1:**
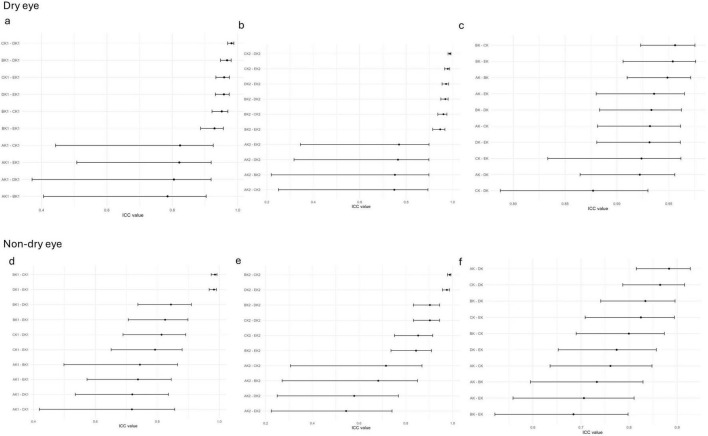
The ICC of corneal curvature measurements among five devices in the non-dry eye and dry eye groups; **(a–c)** The ICC of corneal curvature measurements among all pairwise comparisons of the five devices in the dry eye groups in k1 **(a)**, k2 **(b)**, Astigmatism **(c)**; **(d–f)** The ICC of corneal curvature measurements among all pairwise comparisons of the five devices in the non-dry eye groups in k1 **(d)**, k2 **(e)**, Astigmatism **(f)**; AK1, AK2, AK: K1, K2, Astigmatism values measured by Corneal topography in the Oculus Keratograph 5M; BK1, BK2, BK:K1, K2, Astigmatism values measured by ARK-1a; CK1, CK2, CK: K1, K2, Astigmatism values measured by IOL Master 700; DK1, DK2, DK:K1, K2, Astigmatism values measured by OPD-Scan III; EK1, EK2, EK: K1, K2, Astigmatism values measured by Pentacam.

The ICCs for ARK-1a, IOL Master 700, OPD-Scan III, and Pentacam were close to 1 (> 0.9) in both the dry eye and non- dry eye groups, indicating very high consistency (Figure 1). By contrast, corneal topography consistently produced lower ICC values, suggesting poorer reliability relative to the other devices. Moreover, the confidence intervals in the non- dry eye group were generally narrower than those in the dry eye group, reflecting more stable measurements in patients without dry eye.

Tables 2, 3 present the Bland-Altman analysis results for each device in the non- dry eye and dry eye groups, respectively. In this study, when over 95% of measurement differences between two instruments fall within 95% LoA and the differences are clinically acceptable, the instruments are considered to have good agreement and can be used interchangeably. If less than 5% of differences fall outside 95% LoA but these differences are not clinically acceptable, the agreement is considered poor. This study defined poor agreement as 95% limits of agreement differences exceeding 1.0 D. This means that if the difference between two devices’ measurements exceeds 1.0 D, they are considered to have poor agreement, which may lead to clinical deviations in diagnosis and treatment plans.

**TABLE 2:** The Bland-Altman analysis of corneal curvature measurements from five devices in the non-dry eye group.

Variable 1	A–B	A–C	A–D	A–E	B–C	B–D	B–E	C–D	C–E	D–E
**K1**										
Upper_LOA	2.68	2.81	2.98	2.9	0.57	1.74	1.76	1.79	1.81	0.54
Lower_LOA	−1.51	−1.46	−2.07	−2.22	−0.39	−2.01	−2.26	−2.23	−2.48	−0.77
Width	4.19	4.27	5.05	5.12	0.96	3.75	4.02	4.02	4.29	1.31
**K2**										
Upper_LOA	1.29	1.2	1.9	2.12	0.51	1.37	1.79	1.32	1.67	0.74
Lower_LOA	−2.92	−2.74	−3.66	−4	−0.42	−1.5	−2.03	1.54	−2.01	−0.86
Width	4.21	3.94	5.56	6.12	0.93	2.87	3.82	2.86	3.68	1.6
**Astigmatism**										
Upper_LOA	0.62	0.64	0.8	0.74	0.48	0.72	0.6	0.94	0.74	0.67
Lower_LOA	−0.61	−0.71	−0.65	−0.51	−0.56	−0.57	−0.39	−0.71	−0.44	0.6
Width	1.23	1.35	1.45	1.25	1.04	1.29	0.99	1.65	1.18	1.27

A: Corneal topography in the Oculus Keratograph 5M; B: ARK-1a; C: IOL Master 700; D: OPD-Scan III; E: Pentacam.

**TABLE 3:** The Bland-Altman analysis of corneal curvature measurements from five devices in the dry eye group.

Variable 1	A–B	A–C	A–D	A–E	B–C	B–D	B–E	C–D	C–E	D–E
**K1**										
Upper_LOA	1.85	1.72	1.77	1.71	0.72	0.61	0.81	0.5	0.66	0.6
Lower_LOA	−0.79	−0.7	−0.69	−0.77	−0.76	−0.58	−0.93	−0.43	−0.73	−0.75
Width	2.64	3.42	2.46	2.48	1.48	1.19	1.74	0.93	1.39	1.35
**K2**										
Upper_LOA	0.72	0.78	0.8	0.82	0.74	0.7	0.91	0.44	0.59	0.63
Lower_LOA	−2.07	−2.31	−2.03	−2.03	−0.73	−0.58	−0.77	−0.33	−0.45	−0.61
Width	2.79	2.91	2.83	2.85	1.47	1.28	1.68	0.77	1.04	1.24
**Astigmatism**										
Upper_LOA	0.68	0.66	0.47	0.84	0.71	0.67	0.95	0.59	0.71	0.78
Lower_LOA	−0.86	−0.8	−0.55	−0.76	−0.67	−0.57	−0.69	−0.53	−0.49	0.61
Width	1.54	1.46	1.02	1.6	1.38	1.24	1.64	1.12	1.2	1.39

A: Corneal topography in the Oculus Keratograph 5M; B: ARK-1a; C: IOL Master 700; D: OPD-Scan III; E: Pentacam.

In the non- dry eye group, the narrowest 95% limits of agreement (LOA) in K1 and K2 measurements were found between ARK-1a and IOL Master 700, and between OPD-Scan III and Pentacam (K1: 0.96 and 1.31; K2: 0.93 and 1.6, respectively). This indicates high consistency in corneal curvature measurement among ARK-1a and IOL Master 700. For astigmatism, all device comparisons yielded relatively narrow LOAs (approximately 1.2 in width), suggesting minimal inter-method variation (Table 2).

In the dry eye group, corneal topography generally showed wide LOAs (> 2.0) when compared with the other devices, indicating moderate agreement. Conversely, ARK-1a demonstrated narrower LOAs (ranging from 1.0 to 1.7), reflecting better consistency. Notably, the narrowest LOAs (< 1.0) were observed between IOL Master 700 and OPD-Scan III, indicating superior performance of these two instruments for measuring corneal curvature in dry eye patients (Table 3).

### 3.3 Two-by-two comparison of inter-device measurement differences in the dry eye and non- dry eye groups

**FIGURE 2:**
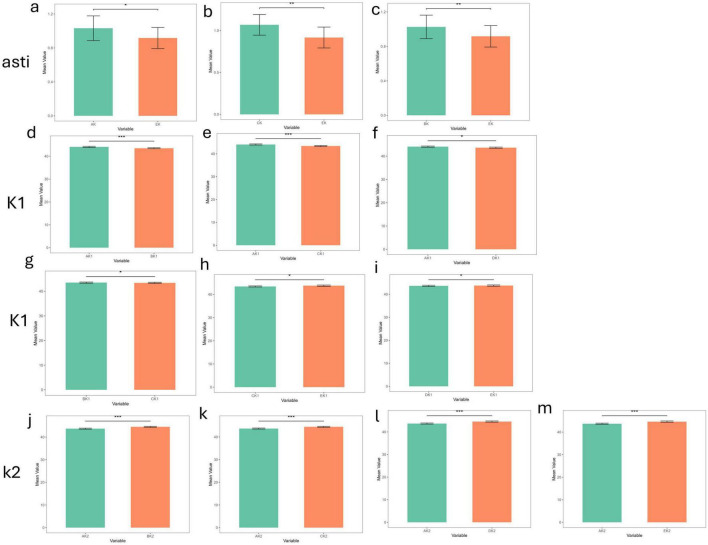
The differences in corneal curvature measurements among the five devices in the non-dry eye group (***p_value < 0.001; **p_value < 0.01; *p_value < 0.05; ns, *p*_value > 0.05). **(a–c)** The differences in corneal curvature measurements among all pairwise comparisons of the five devices in the non-dry eye group in Astigmatism. **(d–i)** The differences in corneal curvature measurements among all pairwise comparisons of the five devices in the non-dry eye group in K1. **(j–m)** The differences in corneal curvature measurements among all pairwise comparisons of the five devices in the non-dry eye group in K2. AK1, AK2, AK: K1, K2, Astigmatism values measured by Corneal topography in the Oculus Keratograph 5M; BK1, BK2, BK:K1, K2, Astigmatism values measured by ARK-1a; CK1, CK2, CK: K1, K2, Astigmatism values measured by IOL Master 700; DK1, DK2, DK:K1, K2, Astigmatism values measured by OPD-Scan III; EK1, EK2, EK: K1, K2, Astigmatism values measured by Pentacam.

**FIGURE 3:**
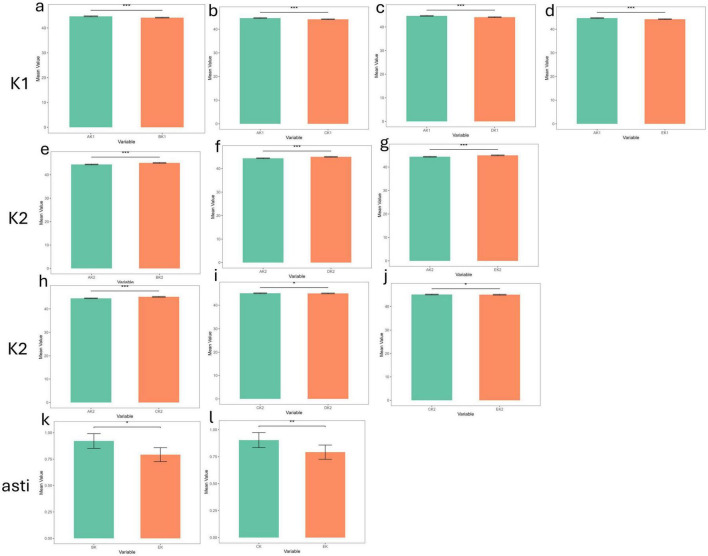
The differences in corneal curvature measurements among the five devices in the dry eye group (***p_value < 0.001; **p_value < 0.01; *p_value < 0.05; ns, *p*_value > 0.05). **(a–d)** The differences in corneal curvature measurements among all pairwise comparisons of the five devices in the dry eye group in K1. **(e–j)** The differences in corneal curvature measurements among all pairwise comparisons of the five devices in the dry eye group in K2. **(k,l)** The differences in corneal curvature measurements among all pairwise comparisons of the five devices in the dry eye group in Astigmatism. AK1, AK2, AK: K1, K2, Astigmatism values measured by Corneal topography in the Oculus Keratograph 5M; BK1, BK2, BK:K1, K2, Astigmatism values measured by ARK-1a; CK1, CK2, CK: K1, K2, Astigmatism values measured by IOL Master 700; DK1, DK2, DK:K1, K2, Astigmatism values measured by OPD-Scan III; EK1, EK2, EK: K1, K2, Astigmatism values measured by Pentacam.

Paired-sample *t*-tests or Wilcoxon signed-rank tests were used to evaluate inter-device differences for K1, K2, and astigmatism (Figures 2, 3). In the non- dry eye group, 13 statistically significant differences were observed in total: 6 for K1, 4 for K2, and 3 for astigmatism. Corneal topography differed markedly (*p* < 0.001) from ARK-1a and IOL Master 700 in K1 measurements and from all other devices in K2 measurements. For astigmatism, Pentacam measurements differed significantly (*p* < 0.01) when compared to ARK-1a and IOL Master 700.

In the dry eye group, 12 statistically significant differences emerged: 4 for K1, 6 for K2, and 2 for astigmatism. Corneal topography showed significant discrepancies (*p* < 0.001) from all other devices in K1 and K2, underscoring potential limitations in its reliability for dry eye patients. Moreover, Pentacam displayed significant differences in astigmatism measurements when compared to ARK-1a and IOL Master 700 (*p* < 0.05, *p* < 0.01), further indicating that its astigmatism measurements may be less consistent in individuals with dry eye. By contrast, IOL Master 700, OPD-Scan III, and Pentacam generally exhibited strong agreement on most parameters, demonstrating high reliability for both clinical and research applications. Although some devices showed statistically significant measurement differences (*p* < 0.05) in the dry eye groups, their clinical significance depends on the magnitude of the differences. If the difference is less than 0.5 D, the impact on clinical decision-making may be minimal; whereas differences exceeding 1.0 D may warrant cautious consideration and should be interpreted in conjunction with other clinical findings.

## 4 Discussion

This study show that dry eye significantly affects the consistency of corneal curvature measurements. In patients with dry eye, careful selection of devices (e.g., IOL Master 700, OPD-Scan III) and consideration of tear film status are crucial for evaluating corneal curvature in applications such as orthokeratology lens fitting, refractive surgery assessment, and pre-cataract surgical planning. In this study, the consistency of five devices was evaluated using both ICC analyses and Bland-Altman plots, and differences were further explored via paired-sample *t*-tests or Wilcoxon signed-rank tests. In general, the non- dry eye group exhibited higher ICC values than the dry eye group, suggesting that dry eye compromised the reliability of measurement outcomes.

The Bland-Altman plots indicated that the 95% limits of agreement were broader in the dry eye group, especially in comparisons involving corneal topography, where the limits exceeded 2.0. These findings underscore the need for caution when interpreting such data in clinical practice. By contrast, in the dry eye group, the IOL Master 700 and OPD-Scan III showed the narrowest consistency ranges (< 1.0), thereby reinforcing their reliability and clinical utility. The principal factor contributing to measurement variability in dry eye patients is the irregular and unstable tear film, which substantially affects the optical quality of the corneal surface (20–23). As a common ocular surface disease, dry eye causes disruptions to corneal and ocular surface properties via several mechanisms. First, reduced tear film stability, a defining feature of dry eye, compromises the optical uniformity of the anterior corneal surface. This phenomenon directly undermines the accuracy of devices reliant on optical reflection or refraction principles (e.g., corneal topography and Pentacam) (24, 25). Second, morphological changes in the corneal epithelium, such as surface roughness and scar formation, further contribute to increased light scattering and optical aberrations (26). This issue is particularly relevant for optical devices with high-resolution requirements. Third, dry eye can introduce astigmatic errors due to localized tear film breakup and optical aberrations (27). Fourth, the fluctuating tear film in dry eye increases variability in repeated measurements from the same device, reducing the overall reliability of results.

Differences in device sensitivity to optical irregularities may further magnify inconsistencies. In this respect, the IOL Master 700 and OPD-Scan III provided more stable and consistent results in the presence of tear film instability, whereas corneal topography, which heavily relies on a smooth tear film surface, was more prone to measurement deviations. Both the IOL Master 700 and OPD-Scan III demonstrated better performance in dry eye patients, likely reflecting their reduced sensitivity to tear film irregularities.

The IOLMaster 700 (Carl Zeiss Meditec AG, Jena, Germany) employs swept-source optical coherence tomography (SS-OCT) for image-based biometry, allowing visualization of the eye’s full longitudinal section. Additionally, it utilizes telecentric keratometry, projecting light onto the cornea to assess curvature, thereby minimizing measurement errors and procedural failures (28–30). This technology enables direct corneal surface measurement by penetrating through the tear film layer, thereby minimizing tear film-related measurement variability. Compared to conventional devices, the IOLMaster 700 exhibits superior reproducibility and measurement accuracy, which align with our experimental results (31, 32).

The OPD-Scan III corneal/refractive analyzer combines a Placido disk-based corneal topography system with a Scheiner-principle subjective aberrometer. It calculates wavefront aberration by scanning retinal grid-pattern light and measuring the deviation between actual and theoretical retinal reflection arrival times. Additionally, the Placido disk projection system captures corneal data by projecting 25–33 concentric rings onto the cornea (33–36). This system compensates for corneal surface irregularities, thereby demonstrating reliable measurement stability in dry eye conditions.

Pentacam and corneal topography, in contrast, are more vulnerable to tear film fluctuations because they rely on optical reflection. Pentacam uses rotating Scheimpflug imaging to acquire three-dimensional corneal and anterior chamber data (37, 38)., but tear film instability introduces light deviation that lowers data accuracy. Corneal topography, based on the Placido ring reflection principle (39, 40), also depends on an intact tear film to maintain measurement reliability. Prior studies suggest that using artificial tears or lubricants, or corroborating corneal measurements with multiple instruments, can help address these challenges in dry eye cases (41–44).

Previous studies have reported lower repeatability in corneal curvature measurements among dry eye populations. Variations in corneal curvature between two measurements in the same patient frequently exceed 0.5 D, with considerably larger discrepancies than those observed in healthy individuals (18). Tear film disruption and corneal surface changes are generally viewed as key contributors to this lack of stability. Indeed, device outcomes often differ in dry eye patients (45). Instruments that rely on optical reflection can be particularly sensitive to tear film instability, whereas devices based on optical coherence tomography exhibit less dependence on tear film condition and may yield more consistent data (46). The present study’s innovation lies in assessing and comparing multiple devices within the same cohort of dry eye patients, thus offering a robust basis for selecting optimal instruments in clinical practice.

From a clinical standpoint, the results offer valuable guidance on assessment and treatment strategies for patients with dry eye. When measuring corneal curvature, the IOL Master 700 or OPD-Scan III are recommended to improve data accuracy and reliability, especially in refractive surgery planning and contact lens fitting. Tear film stability assessment also emerges as critical for device selection and interpretation of results, particularly in severe dry eye. Interventions to improve tear film quality before measurement—such as administering artificial tears—may enhance measurement stability and accuracy. Given the varying performance of devices under dry eye conditions, it is advisable to combine multiple instruments, for example, the IOL Master 700 and OPD-Scan III, to improve diagnostic confidence.

Despite providing important insights, this study has certain limitations. The relatively small sample size restricts the generalizability of the findings. Future research involving larger cohorts will strengthen the statistical power and facilitate analysis of how different degrees of dry eye severity influence measurement consistency and accuracy. Additionally, this study did not investigate the quantitative relationship between tear film quality (e.g., tear volume and thickness) and corneal measurements. Future research could integrate tear film–related parameters to further illuminate the complex interplay between tear film status and corneal optics (47). Finally, continued efforts to develop advanced devices specifically tailored for dry eye and to optimize tear film correction algorithms in existing instruments represent promising avenues for improving clinical outcomes (48).

## 5 Conclusion

The present study systematically assesses the effects of dry eye on various measurement modalities, offering evidence-informed recommendations for clinicians regarding instrumentation selection in dry eye management. This study found that dry eye significantly affects the reliability of corneal curvature measurements, with varying degrees of impact across different measuring devices. Corneal topography and Pentacam performed poorly under dry eye conditions, while IOL Master 700 and OPD-Scan III showed better stability. Therefore, for dry eye patients undergoing refractive surgery planning, contact lens fitting, or cataract preoperative evaluation, priority should be given to measurement results from IOL Master 700 and OPD-Scan III to improve accuracy. In patients with severe dry eye, ensuring tear film stability and addressing corneal surface issues are critical prerequisites for accurate measurements. Employing multiple devices can further improve diagnostic precision and reliability. These findings have significant implications for the personalized diagnosis and management of dry eye in clinical and research contexts.

## Data Availability

The original contributions presented in this study are included in this article/supplementary material, further inquiries can be directed to the corresponding authors.
